# Pain in NMOSD and MOGAD: A Systematic Literature Review of Pathophysiology, Symptoms, and Current Treatment Strategies

**DOI:** 10.3389/fneur.2020.00778

**Published:** 2020-08-21

**Authors:** Susanna Asseyer, Graham Cooper, Friedemann Paul

**Affiliations:** ^1^Experimental and Clinical Research Center, Max Delbrück Center for Molecular Medicine and Charité—Universitätsmedizin Berlin, Corporate Member of Freie Universität Berlin, Berlin Institute of Health, Humboldt—Universität zu Berlin, Berlin, Germany; ^2^NeuroCure Clinical Research Center, Charité—Universitätsmedizin Berlin, Corporate Member of Freie Universität Berlin, Berlin Institute of Health, Humboldt—Universität zu Berlin, Berlin, Germany; ^3^Einstein Center for Neurosciences, Berlin, Germany; ^4^Department of Neurology, Charité—Universitätsmedizin Berlin, Corporate Member of Freie Universität Berlin, Berlin Institute of Health, Humboldt—Universität zu Berlin, Berlin, Germany

**Keywords:** aquaporin 4, headache, myelin oligodendrocyte glycoprotein-antibody-associated disease, neuromyelitis optica spectrum disorders, neuropathic pain, pain, painful tonic spasms

## Abstract

Neuromyelitis optica spectrum disorders (NMOSDs) and myelin oligodendrocyte glycoprotein-antibody-associated disease (MOGAD) are autoimmune inflammatory disorders of the central nervous system (CNS). Pain is highly prevalent and debilitating in NMOSD and MOGAD with a severe impact on quality of life, and there is a critical need for further studies to successfully treat and manage pain in these rare disorders. In NMOSD, pain has a prevalence of over 80%, and pain syndromes include neuropathic, nociceptive, and mixed pain, which can emerge in acute relapse or become chronic during the disease course. The impact of pain in MOGAD has only recently received increased attention, with an estimated prevalence of over 70%. These patients typically experience not only severe headache, retrobulbar pain, and/or pain on eye movement in optic neuritis but also neuropathic and nociceptive pain. Given the high relevance of pain in MOGAD and NMOSD, this article provides a systematic review of the current literature pertaining to pain in both disorders, focusing on the etiology of their respective pain syndromes and their pathophysiological background. Acknowledging the challenge and complexity of diagnosing pain, we also provide a mechanism-based classification of NMOSD- and MOGAD-related pain syndromes and summarize current treatment strategies.

## Introduction

In 1894, Eugène Devic (1858–1930) and his doctoral student Fernand Gault (1873–1936) reported a historical case on a patient with optic neuritis (ON) and myelitis and proposed the name “neuro-myélite optique” for this syndrome. The patient, a 45-year-old woman, was admitted for suspected “neurasthenia,” suffering from disturbed sleep, gastrointestinal symptoms, neuromuscular asthenia, palpitations, and, especially, headache: “*The pain occurs in attacks, both during the day and night. Pain attacks may be long or short, affecting one side of the face and the head, sometimes the right side, mostly the left, but the highest intensity is always at the occipital region: the neck and eyeballs. The pain is sometimes so strong that it causes the patient to cry.”*

One month after admission, the patient suddenly developed acute complete paraparesis and visual loss. It is currently a matter of debate whether the patient suffered from a neuromyelitis optica spectrum disorder (NMOSD) or a myelin oligodendrocyte glycoprotein-antibody-associated disease (MOGAD) (1). Terrible, agonizing, and unbearable pain can arise as an acute or chronic symptom in both pathologies (2–4) (Table 1).

**Table 1:** Characteristics of different pain types.

Pain	Pain is defined as an “unpleasant sensory experience associated with actual or potential tissue damage or described in terms of such damage” (5).
Nociceptive pain	Nociceptive pain occurs as an appropriate encoding of noxious or potentially noxious stimuli. It represents a physiological response that the patient becomes conscious of when nociceptors in bone, muscle, or any body tissue are activated, warning the organism of tissue damage. In response, coordinated reflexes and behavioral responses are elicited (5, 6).
Neuropathic pain	Pain caused by a lesion in, or disease of, the somatosensory nervous system (7).
Acute pain	Physiological response to an acute disease-related damage (8, 9), here NMOSD- or MOGAD-attack related.
Chronic pain	Pain that persists or recurs for more than 3 months (9), (https://www.iasp-pain.org/).

Neuromyelitis optica spectrum disorders (NMOSDs) are rare and, in most cases, relapsing inflammatory diseases of the central nervous system (CNS) (10). In the majority of cases, NMOSDs are associated with serum immunoglobulin G (IgG) autoantibodies (Abs) targeting the astrocyte aquaporin-4 (AQP4) water channel (11, 12). Patients typically suffer from recurrent attacks of severe optic neuritis and/or myelitis (13, 14) and, less frequently, brainstem or brain involvement (15, 16), leading to a diverse range of symptoms, of which severe pain is one of the most frequent and disabling (2, 17–26). Chronic pain occurs in NMOSD with an estimated prevalence between 72 and 86% (2, 18, 27, 28). Over 50% of NMOSD (82% APQ4-Ab positive) patients recalled an increase in pain intensity as the first indicator of a relapse (26) and 25% of patients with NMOSD (82% AQP4-Ab positive) reported pain as their worst symptom, despite also experiencing severe weakness and bladder or bowel dysfunction (26). Neuropathic pain is the most common type of chronic pain with a prevalence of up to over 80% (2, 26), and painful tonic spasms occur with a prevalence of 25–40% (29–32).

MOGAD is another inflammatory autoimmune condition of the CNS, defined by IgG antibodies against conformationally intact myelin oligodendrocyte glycoprotein (MOG) localized on the surface of the myelin sheaths (13, 33, 34). Although there is some phenotypic overlap with AQP4-Ab-positive NMOSD, most researchers consider MOGAD to be a distinct disease entity (35–37). Affected patients may develop any combination of acute disseminated encephalomyelitis, transverse myelitis (long or short), optic neuritis (ON, typically anterior, often bilateral), brainstem pathology often affecting cerebellar peduncles, cranial nerve involvement, and, less frequently, brainstem encephalitis, encephalitis mimicking small vessel CNS vasculitis, and cortical disease with seizures (33, 38–44). Pain is also becoming increasingly recognized as a common and debilitating symptom in MOGAD. However, data in pain in MOGAD are scarce and have to be verified in larger studies: mild chronic pain has a reported prevalence of 86% (2), and severe acute pain in the context of attacks has a prevalence of 70% (38). Furthermore, in addition to the typical retrobulbar pain and/or pain on eye movement, severe and sometimes migraine-like headache can precede visual loss in MOG-Ab-related ON (45, 46), the most-common clinical feature at onset and subsequent relapse (33, 37, 38, 47, 48).

Pain is a very common feature of both diseases and has a higher prevalence and severity compared to multiple sclerosis (MS), where estimates of pain prevalence are ~50% (18, 27, 49). It also has a severe impact on the quality of life of affected patients (2, 18, 26, 27), interfering with physical, emotional, and cognitive aspects of well-being (2, 27, 50), as well as activities of daily life in NMOSD (60–83% AQP4-Ab positive) and MOGAD (2, 18, 26, 27). The higher the pain intensity, the worse the physical and emotional quality of life (2, 51).

The alleviation of pain through careful management and treatment should lead to significant improvement in the quality of life of patients with NMOSD and MOGAD. However, successfully controlling pain is highly challenging in these disorders (2, 26–28), and there is relatively little published literature on therapeutic intervention or treatment of pain as a primary outcome in these patient groups. In order to highlight this and facilitate future research in this critical area, we conduct a systematic review of the current literature on different pain syndromes in NMOSD and MOGAD. Based on this, we propose a mechanism-based classification of NMOSD- and MOGAD-related pain and additionally evaluate current treatment strategies.

## Methods

We performed a search of PubMed (last updated on June 09, 2020), combining neuromyelitis optica or neuromyelitis optica spectrum disorders AND pain, as well as myelin oligodendrocyte glycoprotein AND pain. Additional searches were performed combining neuromyelitis optica and myelin oligodendrocyte glycoprotein, respectively, AND headache or dysesthesia or dystonia or Lhermitte's sign or neuralgia or spasms or spasticity. This search was limited to English language publications and yielded a total of ~200 articles including case reports, original clinical studies, and reviews, which were reviewed by title and abstract for potential relevance to this topic. When the title and abstract did not clearly indicate the degree of relevance to the topic, the article itself was reviewed. Bibliographies of topic-relevant articles were also examined to discover additional references not identified in the primary search. Finally, the authors' personal knowledge of the literature as well as congress contributions to ECTRIMS 2019 were used to supplement the above references.

As the impact of pain in patients with AQP4-Ab-positive and Ab-negative NMOSD is similar, we document both disease types together and report the percentage of AQP4-positive NMOSD patients whenever available. We note that some MOG-Ab-positive patients may have been included in former NMOSD studies. However, the percentage of MOG-Ab-positive patients within groups of Ab-negative NMOSD patients should be low.

## Results

We identified 18 studies evaluating pain in NMOSD (*n* = 17) and MOGAD (*n* = 2, one overlapping with NMOSD) (Table 2).

**Table 2:** Original publications on pain in neuromyelitis optica spectrum disorder (NMOSD) and myelin oligodendrocyte glycoprotein-antibody-associated disease (MOGAD) (listed in chronological order).

**References**	**Patient sample**	**Portion of AQP4-IgG seropositive patients**	**Pain and QoL assessment**	**Imaging data**	**Pain type**	**Pain medication**	**Main findings**
Kanamori et al. (18)	42 NMOSD vs. 51 MS	35/42	SF-BPI SF-36	N.A.	N.A.	N.A.	First study on pain in NMOSD: Pain in NMOSD is more frequent and severe than in MS and has a severe impact on the patients' QoL
Qian et al. (27)	29 NMOSD vs. 66 MS	24/29	MPQ 10-point NRS Interview SF-36	Spinal cord MRI	Retroorbital pain Dysesthetic pain Girdle pain Lhermitte's sign Painful tonic spasms	Tricyclic antidepressants Duloxetine Gabapentin Pregabalin Carbamazepine Lamotrigine Phenytoin Sodium valproate Baclofen Cyclobenzaprine Tizanidine Fentanyl citrate Hydrocodone Hydromorphone Methadone Oxycodone Hydromorphone	First study mentioning specific pain syndromes, including spinal cord MRI and examining medication use: Pain in NMOSD is more frequent and severe than in MS, even after controlling for disability and number of involved spinal cord segments. Pain in NMOSD appears insufficiently controlled by pharmacological interventions
Kim et al. (29)	40 NMOSD vs. 35 MS vs. 42 iATM	34/40	N.A.	Spinal cord MRI	Painful tonic spasms	Carbamazepine Gabapentin Phenytoin	First study on PTS in NMOSD: PTS are a common and relatively specific myelitis-related symptom in NMOSD. PTS most commonly occur during recovery from the first myelitis episode
Usmani et al. (31)	57 NMOSD	1/57	Clinical history	Spinal cord MRI	Painful tonic spasms	Carbamazepine	14% of NMOSD patients had documented typical tonic spasms
Elsone et al. (52)	45 NMOSD	45/45	Clinical history	Spinal cord MRI	Neuropathic pruritus	N.A.	First study on neuropathic pruritus in NMOSD: Neuropathic pruritus seems to be a common but underrecognized symptom of myelitis associated with NMOSD
Pellkofer et al. (28)	11 NMOSD vs. 11 HC	11/11	Interview DN4 NRS QST	MRI	Neuropathic pain	N.A.	First study on NP in NMOSD, evaluating endocannabinoid levels in the serum and somatosensory abnormalities by QST: A total of 91% of the patients suffered from NP within the previous 3 months and 72% reported ongoing pain and decreased QoL at the time of assessment. Plasma levels of 2-AG were higher in NMOSD patients than in HC, suggesting its relevance for central sensitization. QST revealed pronounced mechanical and thermal sensory loss, strongly correlated to ongoing pain suggesting the presence of deafferentiation-induced pain
Zhao et al. (26)	50 NMOSD	41/50	DN4 BPI SF-36	MRI reports	Neuropathic pain	Amitriptyline Duloxetine Gabapentin Pregabalin Carbamazepine Lamotrigine Baclofen Cannabinoids Paracetamol Opiates	Specific exploration of NP and its effect on the QoL. NP was identified in 62% of patients, affecting ADLs. Pain was associated with significant reduction in the SF-36 mental composite score
Mutch et al. (50)	15 NMOSD	9/15	Semistructured interview	N.A.	Neuropathic pain	N.A.	First qualitative study to explore QoL, including pain in NMOSD: NMOSD is a difficult condition to live with due to the unpredictability of relapses and severe disability of visual or spinal symptoms. Poor vision, reduced mobility, bladder dysfunction, and pain affected participants' independence and experience of living with NMOSD
Carnero Contentti et al. (30)	15 NMOSD	15/15	Clinical history	MRI	Painful tonic spasms	Carbamazepine Gabapentin	PTS occur frequently in patients with NMOSD. PTS generally appear a month after a myelitis attack and are associated with extensive cervicothoracic lesions in MRI
Kong et al. (51)	44 NMOSD	29/44	BPI HADS SF-36	N.A:	Pain (not specified)	Codeine Ibuprofen Paracetamol Amitriptyline Duloxetine Diazepam Clonazepam Gabapentin Pregabalin Carbamazepine Oxcarbazepine Baclofen	Pain correlated strongly with quality of life SF-36 physical composite score. Depression highly correlated with pain severity. Pain severity was the most important factor for QoL
Eaneff et al. (25)	522 self-reported NMOSD	N.A.	PatientsLikeMe online questionnaire	N.A.	Pain (not specified)	Duloxetine Gabapentin Pregabalin Baclofen	Moderate to severe fatigue, pain, stiffness, and spasticity limit activities of over 50% of NMOSD patients
Tackley et al. (53)	76 NMOSD	76/76	BPI	MRI	Neuropathic pain	N.A.	Persistent, thoracic cord lesions in AQP4-Ab positive NMOSD is associated with high postmyelitis chronic pain scores, irrespective of number of myelitis relapses, lesion length, and lesion burden
Asseyer et al. (2)	35 NMOSD vs. 14 MOGAD	29/35	painDETECT MPQ SF-36 BDI-II	MRI	Neuropathic pain Headache/neck pain Musculoskeletal pain Spasticity	NSAID Antidepressants Anticonvulsants Opioids	First study exploring pain in MOGAD: Pain is a frequent symptom of patients with MOGAD and has a severe impact on the patients' QoL in NMOSD and MOGAD. Pain is insufficiently alleviated by medication
Liu et al. (32)	230 NMOSD	181/230	Medical records Prospective interviews	MRI	Painful tonic spasms	Carbamazepine Oxcarbazepine Gabapentin Pregabalin Baclofen	22.6% of NMOSD patients experience PTS. Patients with NMOSD and PTS have a higher age at disease onset, higher ARR, and a tendency to experience pruritus. Sodium channel blocking antiepileptic drugs like carbamazepine and oxcarbazepine have higher efficacy than gabapentin in the treatment of PTS
Asseyer et al. (54)	129 MOGAD	No NMOSD	Medical records	MRI	Optic neuritis related headache and orbital/periorbital pain	N.A.	First study on severe headache preceding visual loss in MOG-Ab-related optic neuritis. Florid intraorbital and perioptic inflammation was likely to involve meninges and nociceptive fibers
Hyun et al. (49)	252 NNOSD vs. 248 MS	91/99 who completed PainDetect	PainDetect SF-BPI BDI-II FSS	N.A.	Pain (not specified) Neuropathic pain	N.A.	60% of the NMOSD patients and 34% of the MS patients suffered from current pain. Neuropathic pain was more severe and pain-related interference in daily life was greater in NMOSD patients than in MS patients
Mealy et al. (55)	22 NMOSD	22/22	Self-reported NP attributable to an inflammatory spinal cord lesion NRS	Details n.a.	Neuropathic pain	Antidepressants Anticonvulsants Opioids	First randomized single-blind, sham-controlled trial in NMOSD patients with central neuropathic pain using Scrambler therapy. The median baseline NRS decreased after 10 days of treatment, whereas the median NRS score did not significantly decrease in the sham arm
Wang et al. (56)	38 NMOSD	38/38	BPI	MRI	Neuropathic pain, but not clearly specified	N.A.	First investigation of subcortical structural abnormalities in female NMOSD patients with NP shows significantly smaller hippocampus and pallidum volumes in the patients with NP compared to patients without NP and a significant negative correlation between pain intensity and volumes of the accumbens nucleus and thalamus in patients with NP

*2-AG, 2-arachidonoylglycerol; ADL, activities of daily living; AQP4-IgG, aquaporin 4 immunoglobulin G; ARR, annualized relapse rate; BDI-II, Beck Depression Inventory II; BPI, Brief Pain Inventory; DN4, Douleur neuropathique 4; FSS, Fatigue Severity Scale; HADS, Hospital Anxiety and Depression Scale; HC, healthy control; iATM, idiopathic acute transverse myelitis; MOG-Ab, myelin oligodendrocyte glycoprotein antibody; MOGAD, myelin oligodendrocyte glycoprotein-associated disease; MPQ, McGill Pain Questionnaire; MRI, magnet resonance imaging; MS, multiple sclerosis; NMOSD, neuromyelitis optica spectrum disorders; NP, neuropathic pain; NRS, numeric rating scale; PTS, painful tonic spasms; QoL, quality of life; QST, quantitative sensory testing; SF-36, 36 item short form health survey; SF-BPI, short form Brief Pain Inventory*.

The studies focused on pain without diagnostic specification (18, 25, 51), neuropathic pain (26, 28, 49, 50, 53, 55, 56), one study on neuropathic pruritus (52), painful tonic spasms (29–32), ON-related headache (54), and a description of diverse pain types (2, 27). One randomized single blind sham-controlled trial studied the effect of Scrambler therapy in NMOSD patients with central neuropathic pain (55). All other studies (*n* = 17) were descriptive and non-interventional. Two reviews on pain in NMOSD are available, one focusing on potential mechanisms underlying the pathogenesis of pain in NMOSD and another focusing on the impact of neuropathic pain medication on patients' quality of life (3, 57). Moreover, we included 12 case reports describing pain as part of the patients' symptom complex (4, 58–68). We additionally reviewed studies (*n* = 131) in NMOSD that included pain but where it was not the primary outcome. Where available, we provide the information on the percentage of AQP4-Ab-positive patients of the respective NMOSD cohort. Our review is the first to provide an overview of (1) disease-associated lesion locations in relation to different pain syndromes, (2) different types of NMOSD- and MOGAD-related pain, (3) possibilities to classify acute and chronic pain in NMOSD and MOGAD, and (4) the impact of the currently available immunotherapy on pain.

## Pathophysiological Background of Pain in NMOSD and MOGAD

Inflammatory attacks in the CNS occur in both NMOSD and MOGAD and can lead to acute pain via the release of pronociceptive brain-derived neurotropic factor (BDNF), cytokines and chemokines [interleukin (IL)-1ß, IL-6, IL-17, and tumor necrosis factor (TNF)] (3, 69–71). Cytokine release enhances glutamatergic signaling, the main pronociceptive neurotransmitter in the spinal dorsal horn (3).

### Pathological Substrates of Pain in NMOSD

Under healthy conditions, AQP4 is coexpressed with the excitatory amino acid transporter 2, which enables glutamate uptake by astrocytes. Loss of AQP4 in AQP4-Ab-positive NMOSD may lead to an excessive accumulation of glutamate in the extracellular space. In the context of neuroinflammation and dysregulation of sensory neurons, persistent excessive BDNF, and glutamate concentrations affect vulnerable inhibitory alpha-amino-3-hydroxy-5-methyl-4-isoxazolepropionic acid (AMPA) and gamma-aminobutyric acid (GABA) neurons, respectively (72, 73). The resulting imbalance between excitation and inhibition can then facilitate the development of chronic pain (3, 74, 75). In addition, astrocytes release endocannabinoid 2-arachidonoylglycerol (2-AG), which strongly enhances GABAergic inhibition. Loss of astrocytes in NMOSD leads to 2-AG reduction, likely leading to nociceptive pain and hyperalgesia (28).

Structural cerebral alterations may also affect chronic pain perception in NMOSD. Recently, a study on subcortical abnormalities in female NMOSD patients showed smaller hippocampus and pallidum volumes in patients with neuropathic pain compared to patients without neuropathic pain, as well as a negative correlation between pain intensity and volumes of the accumbens nucleus and thalamus (56). A study on pain-related morphological abnormalities in AQP4-Ab-positive NMOSD described an association of the ventral posterior nucleus (VPN) volume with several measures of pain intensity (76). Both studies suggest that subcortical structures are substantially involved in cognitive, emotional, and modulatory pain processing in AQP4-Ab-positive NMOSD (56, 76).

### Pathological Substrates of Pain in MOGAD

While AQP4-Abs target astrocytes, MOG-Abs bind to myelin-forming oligodendrocytes. Therefore, inflammation in MOGAD primarily causes demyelination with a loss of the microtubule cytoskeleton of oligodendrocytes (13, 77–79). Under healthy conditions, the neuropeptide nerve growth factor (NGF) has a high affinity to bind MOG. Moreover, NGF is part of the nociceptive system: It binds tropomyosin receptor kinase A (TrkA). TrkA is expressed on unmyelinated nociceptive axons of the spinal cord and regulates synaptic strength and plasticity of sensory neurons. Thus, the loss of MOG by antibody-mediated destruction in MOGAD may cause abundant NGF concentrations in the CNS, leading to aberrant sprouting of unmyelinated nociceptive fibers in the posterolateral tract of the spinal cord and hence nociceptive pain (80).

### Lesion Location and Pain in NMOSD

Spinal cord lesions in NMOSD are typically extensive and occur predominantly in the cervical and thoracic spinal cord (17, 81–83). As AQP4 is mainly expressed in the gray matter, lesions concentrate around the central canal, and the adjacent gray matter in the dorsal and ventral horns, as well as in the dorsal root entry zone (84). Ascendant and descendent white matter tracts, including the spinothalamic tract (STT) (52, 85, 86), are affected by severe lesions (87). Tackley et al. report a significant relationship between persistent thoracic myelitis lesions and the severity of neuropathic pain. The presence of cervical lesions, in contrast, were predictive of lower pain scores (53).

In the brainstem, the dorsal medulla oblongata and area postrema have the highest distribution of AQP4 (74, 88). It has been shown that 27% of NMOSD patients with cervical longitudinally extensive transverse myelitis (LETM) showed lesions involving the brainstem (89). Such a distribution could include trigeminal nucleus or periaqueductal gray (PAG) pathology, causing headaches in affected patients (74). The PAG is considered to be a migraine generator and a modulator of headache in NMOSD. Moreover, the hypothalamospinal tract, localized in the dorsolateral medulla, activates the hypothalamus, and the trigeminovascular system. Both regions are considered to be involved in the pathogenesis of headache (90, 91).

Dorsal lesions of the medulla oblongata lead to substance P release, a transmitter that can cause and maintain nociceptive activation of the trigeminal tract nucleus (92). Besides headache, neuropathic pain was also reported more frequently in NMOSD patients with medulla oblongata lesions (85.7% AQP4-Ab positive) than in patients without such lesions (31.8 vs. 11.1% and 65.9 vs. 29.4%) (93). Increased neuropathic pain frequency could be explained by the severe and extensive spinal cord involvement associated with the medulla oblongata (93).

Moreover, AQP4-Ab-positive NMOSD has a predilection to affect the optic nerve (94–96). Astrocytes surrounding the optic nerve express high levels of AQP4, but the unmyelinated optic nerve head also expresses AQP4. Moreover, a high density of retinal astrocytic Müller cells, expressing AQP4, are located in the parafoveal area (97–101).

For a further and more detailed pathophysiological background of possible mechanisms explaining pain in NMOSD, we refer to a review by Bradl et al. (3).

### Lesion Location and Pain in MOGAD

Spinal cord lesions in MOG-Ab-positive myelitis are not always longitudinal and extensive but can still cause sensory symptoms like pain and dysesthesia (38). The axial lesion extension may be crucial for the risk of pain. Depending on the level of the lesion, aberrant nerve fiber sprouting could lead to occipital neuralgia or to more distal neuropathic pain syndromes. Moreover, it has been shown that central neuropathic pain can be induced by oligodendrocyte death and axonal pathology in the spinothalamic tract (102).

The brainstem is a critical region in the pathophysiology of headache. Brainstem lesions are present in up to one-third of patients suffering from MOGAD and could promote the risk for migraine and trigeminal neuralgia (103, 104).

MOG is highly expressed by oligodendrocytes myelinating the optic nerve (105) and is consequently a predominant target in MOG-Ab-related ON. ON-related pain is particularly severe in MOGAD and can present as a migraine-like headache (54). In these cases, severe edema may lead to irritation of the meningeal nerve sheath, which surrounds the optic nerve and contains nociceptive fibers of trigeminal origin (106–108). The trigeminal nerve provides sensory innervation to the ocular and periocular area, and its recurrent branches innervate the intracranial dura, venous sinuses, and cerebral vessels, likely leading to headache (109, 110).

## Types of Pain in NMOSD and MOGAD

Pain can occur during acute attacks and be an indicator of current damage, or it can become a chronic syndrome over the course of the disease. The main pain syndromes in NMOSD and MOGAD comprise ON-related pain, headache, neuropathic pain, and musculoskeletal pain including spasticity, painful tonic spasms, and back pain. We discuss these symptoms in the context of NMOSD and MOGAD below, highlighting any differences between the two diseases where information is available.

### Optic Neuritis-Associated Pain

Optic neuritis is an inflammation of the optic nerve characterized by severe visual loss or blindness associated with ocular pain (111) and occurs in the context of many inflammatory diseases (112–116). ON-related eye pain and pain on eye movement is more common in MOGAD, with reports ranging from 65 to 86% (46, 117, 118), compared to AQP4-Ab-positive ON (28.6–50%) (46, 117) and idiopathic Ab-negative ON (10–46%) (117, 119).

AQP4-Ab-positive ON is typically accompanied by retrobulbar pain often worsened by eye movement (2, 27, 46).

MOGAD-related ON pain seems to be particularly severe, sometimes accompanied by migraine-like headaches that precede the visual deficit (54, 120).

### Headache

Headache is an unspecific but common symptom in NMOSD (2, 74) and has also been described in MOGAD, here mainly associated with optic neuritis (2, 38, 54). It can occur as a first symptom or persist during the disease course (2, 38, 74). NMOSD-related headache can occur as a cervicogenic-like headache (2, 58, 74), neck pain (60, 68), paroxysmal hemicrania (62), or in the context of meningoencephalitis (74, 121). It is typically a mixed pain condition with neuropathic and nociceptive components (74).

#### Cervicogenic-Like Headache

Cervicogenic-like headache is caused by a lesion in or disorder of the cervical spine or soft tissues of the neck. While a few cases presenting with cervicogenic-like headache following myelitis have been mentioned in NMOSD and MOGAD (2, 58, 74), only a single case report has described it in detail: The patient had a left occipital headache spreading to the posterior neck associated with numbness and aching. Response to occipital nerve block was slight, and the headache progressed. MRI revealed an extensive myelitis from the medulla oblongata to the C5 level, a bilateral ocular or prechiasmatic lesion, and suspicious bilateral upper brainstem lesion. Symptoms and MRI pathology improved with steroid treatment (58).

Note that we suggest avoiding the diagnosis of cervicogenic headache in NMOSD and MOGAD in favor of the term cervicogenic-like headache or headache attributed to non-infectious inflammatory diseases (106). Classical cervicogenic headache, in contrast, is caused by a disorder of the cervical spine and its component bony disk and/or soft tissue elements (106).

#### Paroxysmal Hemicrania

Paroxysmal hemicrania is characterized by severe unilateral pain attacks, affecting orbital, supraorbital, and/or temporal regions. The attacks are mostly associated with autonomic features (ipsilateral conjunctival injection, lacrimation, nasal congestion, rhinorrhea, forehead and facial sweating, miosis, ptosis, and/or eyelid edema) (106). We are aware of one case report, describing a patient presenting with paroxysmal hemicrania as first symptom of an AQP4-Ab-positive NMOSD. MRI revealed a lesion extending from the lower medulla oblongata to the cervical cord (C4), possibly involving the spinal nucleus of the trigeminal nerve (62). As in primary paroxysmal hemicrania, indomethacin has been effective in the case of AQP4-Ab-positive NMOSD-related paroxysmal hemicrania (62), but evidence is limited. No reports of paroxysmal hemicrania in MOGAD were identified.

#### Encephalitis-Associated Headache

Meningoencephalitis-like pathology with fever, severe headache, and pleocytosis in the cerebrospinal fluid (CSF) has been reported in both disease complexes, NMOSD and MOGAD (74, 121), most likely due to meningeal inflammation (122, 123).

### Neuropathic Pain

Neuropathic pain is particularly severe (2, 53) and patients typically characterize neuropathic pain as agonizing, shooting, and distressing (57). Neuropathic pain occurs more frequently in NMOSD (83% AQP4-Ab positive) than in MOGAD (80 vs. 40%) (2, 27). It can occur as an early myelitis-related symptom or develop during the disease course (3, 50, 53). Medication is currently not sufficient to control neuropathic pain (2, 27), particularly in patients with AQP4-Ab-positive NMOSD (51). A higher dosage of pain medication was not associated with being free of pain but rather with greater cognitive dysfunction and fatigue (27).

Neuropathic pain can be permanent or intermittent like Lhermitte's sign (27, 81, 124) and is localized either on the extremities or on the trunk, the latter often defined as a girdle sensation (18, 26, 28, 124, 125).

Lhermitte's sign is often painful and occurs in 35–60% of AQP4-Ab-positive NMOSD patients (27, 81, 124). It is defined as a brief, electric-shock-like sensation that runs from the back of the head down the spine, provoked by inclining the neck forward (124). It has been proposed that Lhermitte's sign occurs because demyelinated sensory fibers are hyperexcitable to percussion or elongation (124).

The girdle sensation describes an often burning sensation on the skin, localized with an extension of three or four dermatomes between T3 and T11 (124). It has been reported in 45.8–69% of NMOSD (83% AQP4-Ab positive) patients and can sometimes be misdiagnosed as acute abdomen (27, 124). Schöberl et al. describe an AQP4-Ab-positive NMOSD patient presenting with typical area postrema syndrome who developed an unusual painful segmental erythema resulting from a dorsolateral spinal cord lesion at C6/7 level. A dysregulated A-beta-fiber-evoked vasodilation has been discussed as a possible underlying pathophysiological mechanism (126). Pelvic pain has been reported to occur as an unusual presentation of AQP4-Ab-positive NMOSD, following a lesion of the conus medullaris (61).

Brainstem pathology can also cause neuropathic pain syndromes like trigeminal (2, 16, 74, 127) and occipital neuralgia (2, 128) in NMOSD and MOGAD. Trigeminal neuralgia is defined by pain in the area of the trigeminal innervation (usually V3 and/or V3 division). It is typically characterized by paroxysmal, sudden attacks of short severe stimulus-triggered and electric-like pain episodes (74). Interestingly, NMOSD patients with trigeminal neuralgia rarely show MRI pathology affecting the trigeminal root entry zone (129). It has been discussed whether or not a dual mechanism including pontine plaques and consecutive neurovascular compression may contribute to the pathophysiology (74). Neuropathic pruritus has also been described following brainstem and spinal cord lesions (52). Pruritus is defined as “an unpleasant cutaneous sensation provoking the desire to scratch.” Neuropathic pruritus is caused by affected pruritogenic neurons in the absence of a pruritogenic substance (52). Neuropathic pruritus associated with myelitis has been observed in 27.3% of ACQP4-Ab-positive NMOSD patients, either as a first symptom or a few days after the onset of other myelitis-related symptoms. It has a sudden onset of high intensity with a duration from seconds to minutes, associated with superficial sensory deficits and/or pain. It can occur on the trunk, the extremities, or the occipital region of the head (52). An inflammation-related demyelination involving second-order itch neurons in the dorsal horn of the spinal cord has been discussed as an underlying pathophysiological mechanism. The role of brainstem lesions affecting the spinal nucleus of the trigeminal nerve or periaqueductal pathways has also been discussed (52, 130).

Very few studies have focused on neuropathic pain in MOGAD. Lhermitte's sign (38, 45), band-like girdle sensations (131), trigeminal and occipital neuralgia, and neuropathic extremity pain (2, 38) have been mentioned but have so far not been studied in detail. Myelitis in MOGAD may have a better tendency to recover (83) and therefore cause less severe central neuropathic pain syndromes than in NMOSD.

#### Peripheral Nervous System-Related Neuropathic Pain

Some cases of possible peripheral nervous system (PNS) involvement in NMOSD have been published. Painful, flaccid paralysis (63), lumbosacral myelitis (132), clinical and electrophysiological second motor neuron involvement (133), and peripheral neuropathy (134, 135) have been described, and radicular pain has been reported to occur in up to 33% (81, 136). Recently, a few cases with PNS involvement in MOGAD have been described. Cranial nerve involvement, brachial neuritis, multifocal neuropathy, migratory paresthesia, myeloradicular symptoms, recurrent limb paresthesia, and pain have been mentioned (41, 64, 137, 138). As described above, the inflammatory process in the CNS could trigger an immune cascade targeting myelin-specific antigens in the nerve roots. Alternatively, low quantities of MOG may be expressed in the human peripheral myelin and the Schwann cells, as previously described in rodents and primates (64, 138, 139). However, current data are too scarce for pathophysiological conclusions. At present, we can only infer that PNS involvement should not prevent clinicians from investigating the presence of MOG- and AQP4-IgG Abs.

### Spasticity and Painful Tonic Spasms

Spasticity is defined as “disordered sensorimotor control resulting from an upper motor neuron lesion, presenting as intermittent or sustained involuntary activation of muscles.” At the patient level, it can be defined as an “unusual tightening of muscles that feels like leg stiffness, jumping of legs, a repetitive bouncing of the foot, muscle cramping in the legs or arms, legs going out tight and straight or drawing up” (140). More than 50% of NMOSD patients are reported to suffer from moderate to severe spasticity (25), but very little is known about spasticity in MOGAD (1, 38).

Painful tonic spasms are defined as paroxysmal, recurrent muscle spasms in one or more limbs and/or the trunk, lasting seconds to minutes, accompanied by intense pain and dystonia (29, 30, 65). Several case reports and small series describe PTS in NMOSD (18, 29, 29–31, 65–67, 136, 141–144), but no reports were identified mentioning PTS in MOGAD. Abboud et al. reported that all patients with tonic spasms had associated neuropathic pain (145). PTS and pain occur more frequently in NMOSD than in MS (18, 29), and PTS-associated myelitis in AQP4-Ab-positive NMOSD has been described with a specificity of 98.7% compared to MS (143). Kim et al. showed that transverse myelitis at disease onset, but not optic neuritis, was predictive of future occurrence of PTS. PTS develop mainly during recovery from the first myelitis attack within a mean of 48 days without occurrence of new MRI lesions (3, 29, 30). A spinal cord syndrome with paroxysmal tonic spasms may be particularly suggestive for NMOSD (29, 81). PTS may occur following the loss of inhibitory motor neurons in the central gray matter of the spinal cord (142). Abnormal demyelination can cause ephaptic transmission between the tracts causing spasms (65). As nerve damage does not affect somatosensory pathways, PTS are not considered to be of neuropathic origin (146).

### Back Pain

Like headache, back pain is an unspecific syndrome but occurs frequently in NMOSD and MOGAD (1, 38, 131, 147, 148). It can emerge in the context of myelitis following radiculitis as described above but is often a mixed syndrome including central and peripheral neuropathic as well as nociceptive pain components. Malposition and axial instability following paresis or spasticity, reduced mobility with wheelchair dependence, or long-term corticoid therapy leading to osteoporosis are important secondary aspects to consider in these disorders and can enhance pain, especially back pain (5).

### Comorbidity-Related Pain

Up to 45% of patients with NMOSD and ~10% of patients with MOGAD suffer from autoimmune comorbidities (13), including connective tissue disease, dermatomyositis, rheumatoid arthritis, Sjoegren's syndrome, systemic lupus erythematodes, vasculitis, and myasthenia gravis (2, 51, 148–155), which can themselves be associated with pain (156). A careful diagnostic workup is necessary to detect potentially overlapping pathologies.

## Additional Factors Associated With Pain in NMOSD and MOGAD

Women are more often affected by autoimmune diseases than men, with a female/male ratio of up to 10:1 in NMOSD and, depending on the geographic region, between 1.1:1 and 3:1 in MOGAD (13, 157). However, no sex differences have been found concerning pain prevalence or intensity (26). Mixed results have been found regarding the correlation between pain intensity and age (18, 26). Severe overall disability, measured by the expanded disability status scale (EDSS), has been identified as a risk factor for more severe pain (27) and increasing disability scores correlated with pain intensity in NMOSD (83 and 66% AQP4-Ab positive) (27, 51). Moreover, an association of depression, fatigue, and NMOSD (66–83% AQP4-Ab positive) as well as MOGAD has been shown in several studies (2, 19, 27, 51, 158). Depression and pain are known to interact, and one cannot be certain whether depression enhances pain, occurs in response to pain, or both (159).

## Classification of Pain in NMOSD and MOGAD

The International Association for the Study of Pain (IASP) defines pain as an “unpleasant sensory experience associated with actual or potential tissue damage or described in terms of such damage” (https://www.iasp-pain.org/). We propose a classification for pain in NMOSD and MOGAD (Table 3), which is similar to a previously provided MS-related pain classification (146). Our aim is to present a structure providing

1) the time course of pain development, to distinguish
  - pain as a warning signal of acute damage
  - pain as a self-sustaining chronic syndrome
2) the underlying pathophysiological mechanisms, to distinguish
  - ON-related pain
  - headache
  - neuropathic pain
    - intermittent (episodic), e.g., trigeminal and occipital neuralgia, Lhermitte's sign
    - permanent (continuous), e.g., pain in the extremities
  - spasticity and painful tonic spasms
  - mixed pain, e.g., back pain
  - comorbidity-related pain
3) a reference for specific treatment strategies
4) a framework to generate future research hypotheses.

**Table 3:** Classification of pain in neuromyelitis optica spectrum disorder (NMOSD) and myelin oligodendrocyte glycoprotein-antibody-associated disease (MOGAD).

**Pain condition**	**Examples**
**Acute pain**	
ON-related pain	Retro- or periorbital pain, increased by eye movement. In MOGAD: associated headache possible.
Headache	Cervicogenic-like headache, paroxysmal hemicrania, encephalitis-related headache
Neuropathic pain	Myelitis-related neuropathic pain: dysesthetic extremity pain, neuropathic pruritus, girdle sensation, pelvic pain
**Chronic pain**	
Intermittent neuropathic pain	Lhermitte's sign, trigeminal neuralgia, occipital neuralgia
Permanent neuropathic pain	Dysesthetic extremity pain, neuropathic pruritus, girdle sensation, pelvic pain
Spasticity-related pain	Leg stiffness, muscle cramping in the legs or arms
Painful tonic spasms	Paroxysmal, recurrent muscle spasms in one or more limbs and/or the trunk
Back pain	Multifactorial pathology including nociceptive and neuropathic aspects, e.g., following spasticity

Of note, acute and chronic pain syndromes can overlap. For efficacious treatment, a detailed medical history is necessary.

## Treatment of Pain in NMOSD and MOGAD

Despite the use of multiple medications, pain is currently not sufficiently managed in NMOSD or MOGAD (2, 26–28), and there is relatively little published literature on therapeutic intervention or treatment of pain as a primary outcome in these patient groups. Three studies on immunosuppressive treatment in AQP4-Ab-positive NMOSD have shown promising results when examining pain as a secondary outcome: two in patients treated with the humanized monoclonal IL-6 antibody tocilizumab (125, 160, 161) and one in patients treated with low-dose mycophenolate mofetil (MMF) (162). One study on the positive effect of Scrambler therapy for the treatment of neuropathic pain in NMOSD was identified (55). No studies were found investigating pain treatment in MOGAD. We provide an overview of current strategies for relapse-related treatments and effects of immunosuppressive treatment focusing on acute and chronic pain, respectively. We additionally give a general overview on the management of chronic neuropathic pain, spasticity-related pain, and painful tonic spasms, although these are not specific to NMOSD or MOGAD.

### Attack-Related Treatment

Attack-related treatment aims to reduce pain by reducing the destruction of the CNS. In NMOSD, as well as in MOGAD, acute attacks are usually treated with 1,000 mg intravenous methylprednisolone (IVMP) for 3–5 days (163). Prompt treatment initiation should also be considered in patients who present with pain as their only symptom, in order to avoid rapid progression and attack-related disability (148). Of note, attack-related disability can cause the development of secondary pain, e.g., paresis- and malposition-related pain, reflecting attack-independent disease progression. Rapid corticoid therapy showed prompt recovery from pain in NMOSD (120), and Jarius et al. showed nearly complete recovery in 50% of IVMP-treated MOG-Ab-related attacks (38). In cases of poor outcome, IVMP therapy can be increased to 2,000 mg/day. Such a high-dose IVMP therapy, however, seems to be less effective than plasma exchange or immunoadsorption (13, 164–166). Especially in isolated myelitis, it has been shown that clinical response to immediate plasma exchange (PLEX) was better compared to high-dose steroid therapy (166). This could be of relevance in the treatment of patients presenting with neuropathic pain.

Bradl et al. suggest a multidrug treatment at an early disease stage to limit the previously discussed complex interactions of proinflammatory and pronociceptive molecules in order to avoid pain instauration. They propose an approach similar to the treatment for traumatic brain injury, involving minocycline, peroxisome proliferator-activated receptor agonists, cell cycle inhibitors, statins, and progesterone (3). However, currently, there are no data on possible preventive effects on pain development in NMOSD or MOGAD in this regard.

### Effect of Immunomodulatory Treatment on Pain in NMOSD and MOGAD

Immunosuppressive therapy is essential to reduce disease activity and to avoid relapses in NMOSD and MOGAD, again with the aim to reduce the risk of future CNS damage. Up to now, although recommendations for treatment of NMOSD are available, these are not based on a high level of evidence (163, 167, 168). It is strongly recommended that patients suffering from AQP4-ab-positive NMOSD should receive immunotherapy after the first attack. Currently used preventative treatments in NMOSD include prednisone, azathioprine, rituximab, MMF, intravenous immunoglobulins (IVIGs), eculizumab, and methotrexate (163, 168, 169). Data on the efficacy of IVIG, however, are scarce (13). Of note, in Canada, the USA, and Europe, Eculizumab is currently the only approved therapy for the treatment of NMOSD, and all other medications are used off-label and empirically. In clinical trials, the positive effects on relapse rates of inebelizumab and satralizumab NMOSD have been described (13, 160, 169–173). Satralizumab has shown no benefit on pain intensity in two phase III studies (171, 174), and no data on pain are available for eculizumab and inebilizumab (169, 170, 173). As mentioned above, tocilizumab and MMF in contrast have shown positive effects on pain in AQP4-Ab-positive NMOSD. Still, evidence has to be proven in prospective studies focusing on pain as a primary outcome.

Tocilizumab is an antibody against IL-6, a major cytokine involved in NMOSD pathophysiology (175). It has been shown that NMO-IgG binding to AQP4 on astrocytes selectively induces internalization of AQP4 and production of IL-6 (70), which is thought to enhance the survival time of plasmablasts, which generate anti-AQP4 antibodies (71). IL-6 is a pronociceptive cytokine, which plays an important role in the development of neuropathic pain (176). Treatment with tocilizumab leads to reduced immunological activity, as well as neuropathic pain reduction (59, 125, 160), and should therefore be considered in patients at risk for neuropathic pain.

MMF is an immunosuppressant inhibiting the inosine monophosphate dehydrogenase. Consequently, the synthesis of guanosine nucleotide is reduced, which leads to an inhibition of B- and T-lymphocyte proliferation. MMF can be administered in both NMOSD and MOGAD, in the latter preferably in combination with steroids (13, 162). MMF reduces immunological activity and has a positive effect on pain intensity in AQP4-Ab-positive NMOSD patients (162). Unfortunately, the type of pain was not defined in this study.

Of note, pain can occur as a side effect of some immunosuppressive therapy. Eculizumab, inebilizumab, MMF, and rituximab can lead to headache, MMF can cause abdominal pain (13, 120, 170), and inebilizumab can cause back pain, extremity pain, and chest pain (173).

It has to be kept in mind that NMOSD and MOGAD are distinct nosologic entities regarding their underlying pathogenesis (36). In MOGAD, long-term immunotherapy is often considered and recommended only after a second attack in light of the presumably high proportion of monophasic cases and the overall good recovery. Empirical data suggest oral steroids as mainstay of treatment, and slow tapering is crucial to avoid recurrence of disease activity (33, 177, 178). In contrast to NMOSD, the efficacy of rituximab in MOGAD is controversial. Two recent studies showed that up to 45% of the patients under rituximab treatment still relapsed, despite an effective biological effect of rituximab. Consequently, memory B-cell depletion seems to be unable to prevent relapses in a subset of patients suffering from MOGAD (179, 180). Currently, a long-term treatment with intravenous immunoglobulins, or in some cases with methotrexate, may be preferred (13, 38). Like in NMOSD, treatment of MOGAD with classical MS drugs should be avoided, as they can worsen the disease course (181). Up to now, no treatment guidelines with high grade evidence are available for the treatment of MOGAD, and all medications are used off-label and empirically. Of note, none of the immunotherapies have been studied with regard to a potential effect on pain in MOGAD.

### Symptomatic Pain Treatment

Symptomatic therapies aim to treat pain. Of note, the efficacy of the following treatment strategies have not been specifically demonstrated in NMOSD or MOGAD-related pain.

#### Neuropathic Pain

Based on the pathophysiological course of neuropathic pain development and the mechanisms of action, Bradl et al. suggest inducing pharmacological inhibition of glutamatergic signal transduction early in the disease course, e.g., by *N*-methyl-d-aspartate (NMDA)-receptor blockade with low-dose ketamine or memantine. In patients with established lesions and reduced antinociceptive inhibition in advanced disease stages, Bradl et al. propose medication with GABA agonists, e.g., baclofen, and monoamine reuptake inhibitors (3). However, evidence on its effects is limited, and none of these agents are routinely used clinically (3, 182).

Regarding the current state of pain research, multidisciplinary care in combination with tricyclic antidepressants (TCAs), serotonin norepinephrine reuptake inhibitors (SNRIs), gabapentanoids, and tramadol are the most effective options to treat central neuropathic pain (7, 183–185). Depending on the type of medication, a 3–8-week trial is recommended to evaluate its effect. If no significant pain relief can be achieved, the dosage should be adjusted if the medication is tolerated by the patient. In a second step, alternative medication, combination therapy, or evaluation for neurostimulation may be considered (182, 186).

For the effect of medical neuropathic pain treatment on the patients' self-reported quality of life, we recommend the review by Mealy et al. (57).

##### First-line therapy

Tricyclic antidepressants like nortriptyline and amitriptyline show pain-relieving effects by inhibiting serotonin and noradrenaline reuptake (5, 182, 183, 187–189). Nortriptyline and amitriptyline should be started with a daily dose of 10–25 mg per os (p.o.) and increased to a maximal daily dose of 150 mg. Side effects comprise falls, cardiac arrhythmias, orthostatic dysregulation, urinary retention, and dry mouth, and occur especially in elderly people (182, 184, 187, 190, 191).

Serotonin and norepinephrine reuptake inhibitors (SNRIs) like duloxetine and venlafaxine enhance monoamine neurotransmission in the descending inhibitory spinal pathways, resulting in decreased sensation of pain (183–185, 187–189). SNRIs showed positive effects on neuropathic pain in MS but without a corresponding positive effect on the patients' quality of life. Duloxetine should be started with a daily dose of 30 mg p.o. and increased to a maximal daily dose of 60 mg. Venlafaxine should be prescribed with an initial daily dose of 37.5 mg p.o. and escalated to a maximal daily dose of 200 mg. Side effects include mainly renal and liver pathology (7, 57, 182–185, 187, 191).

Gabapentanoids are anticonvulsant drugs, including gabapentin and pregabalin. These drugs inhibit neurotransmitter release in the dorsal horn of the spinal cord by blocking presynaptic alpha-2-delta calcium channels, leading to pain relief. Gabapentin has been shown to effectively decrease pain intensity and improve quality of life of patients suffering from neuropathic pain after spinal cord injury. Gabapentin dosage should also be increased slowly, starting with a daily dose up to 600 mg p.o., and escalating to a maximum daily dose of 3,600 mg. Pregabalin should be initiated with a daily dose of 150 mg p.o. and escalated to a maximal daily dose of 600 mg. Effective pain release by gabapentanoids should be evaluated after a 4–6-week period with 2 weeks at the maximum tolerated dose. Side effects include mainly renal pathology (57, 182–184, 187–190, 192, 193).

For the treatment of trigeminal neuralgia, carbamazepine is considered to be a first-line therapy (184). Carbamazepine can be induced with a daily dose of 200–400 mg. Slowly increasing the dosage by 50 mg/day can be continued up to 600–1,200 mg/day. Especially in elderly people, the tolerance of dosages above 600 mg/day is often poor with important motor and sedative side effects. Apart from the treatment of trigeminal neuralgia, carbamazepine is considered a third-line therapy for neuropathic pain (5, 194, 195).

Medication of first-line treatment should be trialed over an average time period of 4–6 weeks. If sufficient pain relief is not achieved, progression to the next medication or next line of treatment should occur (182, 184, 187, 189, 191).

##### Second-line therapy, including tramadol and combination therapy

Most guidelines consider tramadol as a second-line therapy (182, 189–191, 196). However, for acute neuropathic pain and intermittent exacerbations of neuropathic pain, it is considered first-line medication (182, 189, 191). Tramadol primarily acts as a weak μ-opioid agonist and inhibits serotonin and norepinephrine reuptake. One study on neuropathic pain after spinal cord injury showed a positive effect of tramadol, in addition to stable regimen (57).

Tramadol should be started with a daily dose of 50 mg p.o. and escalated to a maximal daily dose of 400 mg. Side effects comprise seizure disorder and renal impairment, notably in the elderly (182).

Combination therapy is common in the treatment of neuropathic pain. The patient should be closely observed due to an increased risk for side effects (182, 187).

Cannabinoids have shown a positive impact on pain, sometimes additionally improving quality of life (5, 57). Cannabinoids bind to the presynaptic cannabinoid receptor, reducing calcium influx from voltage-gated calcium channels, and hyperpolarization. Consequently, cellular excitability decreases. However, cannabinoids are currently only licensed in Canada, Israel, and New Zealand for the treatment of neuropathic pain and the safety profile remains a matter of debate (5, 57).

##### Third-line therapy

For patients who do not tolerate first- or second-line therapy or do not benefit from adequate pain relief, medication with serotonin-specific reuptake inhibitors (SSRIs), anticonvulsants such as lamotrigine, carbamazepine, topiramate, sodium valproate, and NMDA antagonists, as well as tapentadol, can be considered in a specialized setting. Tapentadol is a newer weak μ-opioid agonist, and strong norepinephrine reuptake inhibitor that does not affect serotonin reuptake. Due to its increased potency compared to tramadol, it is currently considered third- or fourth-line treatment. Evidence grades of third-line treatments are currently relatively low (182, 184, 187–189, 191).

##### Fourth-line therapy

Neuromodulation, including intracranial stimulation, spinal cord stimulation, high-frequency and burst spinal cord stimulation, and dorsal root ganglion stimulation, is considered to be fourth-line treatment before starting medication with long-term opioids (55, 182). As mentioned above, one phase II study has shown a positive effect of Scrambler therapy for the treatment of neuropathic pain in 22 AQP4-positive NMOSD patients (55). Scrambler therapy is non-invasive technology with Food and Drug Administration (FDA) 510(k) approval for acute, chronic, and postoperative pain. Scrambler is a transcutaneous electric nerve stimulation (TENS) technique that stimulates ascending peripheral C-fibers. It aims to modify nociceptive pain by reorganizing maladaptive signaling pathways in the sensory cortex (197). The trial showed pain reduction from a median baseline numeric rating scale (NRS) pain score of 5.0–1.5 after 10 days of treatment. The median NRS score did not significantly decrease in the sham arm (55). Currently, the lack of clear guidelines regarding the frequency and stimulation amplitude necessary to achieve sufficient pain reduction currently limits the use of TENS (57, 198, 199). A phase III study would be necessary to prove the effect of Scrambler therapy on pain, reduction in analgesic medication, and QoL in a larger NMOSD cohort (57, 198, 199).

##### Fifth-line therapy

Low-dose opioid medication to treat permanent neuropathic pain is currently considered as fourth- and fifth-line treatment, if appropriate conservative pharmacological and interventional management (neurostimulation) has failed (182). Opioids bind to an opioid receptor, inhibit adenylyl-cyclase, lead to neuronal hyperpolarization, and decrease neuronal excitability. However, opioids are considered to have a limited efficacy on neuropathic pain, and safety concerns require strict monitoring (7). Combination therapy of gabapentin and opioids provided better neuropathic pain relief than gabapentin or opioids alone but was associated with increased levels of adverse events (182).

##### Other pharmacological options

Baclofen has shown a positive effect on myelitis-related neuropathic pain in MS patients after intrathecal administration (5–1,200 μg/day). However, baclofen is currently not licensed for the treatment of neuropathic pain but rather indicated for medical treatment of spasticity (5, 146). Some patients may benefit from its positive overlapping effects.

#### Spasticity-Related Pain

Spasticity can cause discomfort and stiffness and lead to pain, e.g., back pain (194). Management should be patient focused and target function rather than aiming to reduce the degree of spasticity. Effectively reduced spasticity can accentuate profound underlying weakness, which contributes to the disability and potential complications of malposition. To avoid complications like pain, early treatment of spasticity should emphasize self-management strategies, education, and physiotherapy (200).

Oral pharmacological agents most commonly used to treat spasticity are baclofen, tizanidine, benzodiazepines, dantrolene, and gabapentin (3, 200). If oral medication does not reach the sufficient effect, antispastic agents such as botulinum toxin, intrathecal baclofen, phenol, and cannabinoids can be administered (200, 201). A positive effect on both spasticity and pain has been shown for baclofen, gabapentin, botulinum toxin, and cannabinoids (194, 202).

##### Oral baclofen

Baclofen is a derivate of ɤ-aminobutyric acid (GABA), which can cross the blood–brain barrier to a limited extent. GABA is a major inhibiting CNS transmitter of impulse transmission, and baclofen is thought have an antispastic effect through the inhibition of reflex neurological transmissions in the spinal cord. Baclofen should be administered starting with a daily dose of three times 5 mg p.o. and increased to a maximal daily dose of 80–100 mg. Common side effects include drowsiness, weakness, paresthesia, and dry mouth (194).

##### Intrathecal baclofen

As oral baclofen crosses the blood–brain barrier only to a small extent, the administration of baclofen directly to the site of antispastic action into the spinal canal improves efficacy and reduces potential side effects. A programmable infusion pump allows a continuous supply of the drug. Dosage has to be titrated over time. Long-term dosage used in MS-related spasticity ranged from 21 to 648 μg/day (194).

##### Botulinum toxin

The effect of botulinum toxin (botox, dysport) is to inhibit acetylcholine release at the neuromuscular junction. Despite permanent blockade, the clinical effect of botulinum toxin injections is reversible because of nerve sprouting and muscle reinnervation (200). The total dosage of botox should be ≤200 units and the dosage at one site ≤50 units. Dysport should be started with a total dosage of 500 units per patient. Depending on the clinical response, the dosage of dysport can range from 250 to 1,000 units (200).

##### Gabapentin

Gabapentin is increasingly used as first-line treatment for spasticity, most particularly since it is licensed for neuropathic pain. Its mode of action, administration, and side effects are described in the section of first-line neuropathic pain treatment.

##### Cannabinoids

The medical use of cannabinoids remains controversial. The two most studied cannabinoids in cannabis are tetrahydrocannabinol (THC) and cannabidiol (CBD). THC is the most psychoactive substance and CBD is the major non-psychotropic substance in cannabis. Two cannabinoid receptors, CB1 and CB2, have been identified. CB1 receptors are located in the CNS and on peripheral nerves. CB2 receptors are found on the cells of the immune system. Evidence for successful treatment of both spasticity and pain in MS is available for nabiximols (trade mark: sativex oral spray), oral cannabis extract (OCE) (trade mark: cannador), and synthetic THC (trade mark: dronabinol, nabilone). OCE and THC, however, show only patient-reported spasticity reduction but were not found to be effective to reduce objective measures of spasticity (201, 202).

Nabiximols is a natural cannabis extract with a 1:1 ratio of THC and CBD activating CB1 and CB2 receptors. Nabiximols is available as oromucosal spray with 2.7 mg of THC and 2.5 mg of CBD per actuation (202). Nabiximols has also shown good efficacy for painful tonic spasms (202).

Cannador is a natural cannabis extract with 2.5 mg of THC and 1.25 mg of CBD per capsule and is currently only available in a research setting in Europe. Dronabinol and nabilone are currently not licensed for the treatment of spasticity and pain (202).

##### Painful tonic spasms

In addition to physiotherapy, most frequent medications used to treat PTS are sodium-channel-blocking antiepileptic agents such as carbamazepine, oxcarbazepine, gabapentin, clonazepam, and phenytoin sodium, as well as benzodiazepines, barbiturates, baclofen, and cannabinoids (3, 5, 31, 202, 203). It has been reported that topiramate at a daily dose of 400 mg can lead to the alleviation of PTS in AQP4-Ab-positive NMOSD (67) and one AQP4-Ab-positive NMOSD case with a favorable response to levetiracetam has been described (142). The highest efficacy for NMOSD-related PTS has been reported for carbamazepine, oxcarbazepine, and gabapentin (29, 32), with carbamazepine and oxcarbazepine outperforming gabapentin (32). These recommendations refer to a daily dose of 600–1,200 mg of oxcarbazepine and 100 mg three times a day of carbamazepine compared to 300 or 600 mg three times a day of gabapentin (32). Carbamazepine and oxcarbazepine act as voltage-gated sodium channel blockers and decrease neuronal excitability (32). Considering the emergence of important side effects of carbamazepine, oxcarbazepine has been recommended as a first-line treatment, preferably in combination with antispastic medication or antidepressants such as baclofen, pregabalin, or duloxetine (32).

Side effects of carbamazepine comprise ataxia, dizziness, somnolence, leukopenia, Steven–Johnson syndrome, and hyponatremia (204). In MS, carbamazepine can lead to a reversible exacerbation of neurological symptoms (205). Oxcarbazepine is better tolerated and safer than carbamazepine, especially with respect to CNS secondary side effects (ataxia, somnolence, and dizziness) and interaction with other medications (206). Side effects are often resolved after the titration period or with dosage adjustment. Frequently reported adverse effects include dizziness, headache, nausea, somnolence, fatigue, vomiting, back pain, diarrhea, tremor, skin rash, and blurred vision (206).

### Non-pharmacological Treatment

Pain is more than just an unpleasant physical sensation. It can comprise emotional, social, and spiritual suffering. Therefore, treatment strategies should not only directly target pain relief. Besides psychotherapy or behavioral therapy, exercise programs for physical reconditioning, relaxation techniques, and patient education should be considered to target functional, affective, social, and spiritual consequences affecting the patients' quality of life (182, 207, 208). Currently, pain syndromes in NMOSD and MOGAD are insufficiently controlled by medication, and multidrug therapy has been associated with worse fatigue and depression (2, 27, 209). Therefore, future studies should explore the efficacy of a multimodal and multidisciplinary approach of pain management (27).

## Summary

Pain is a very frequent symptom in NMOSD and MOGAD and has a prevalence of over 80% with a severe impact on the quality of life of affected patients. Pain syndromes differ between NMOSD and MOGAD and can be an indicator for the respective disease type. Acute pain syndromes like retro-orbital pain, headache, or dysesthetic pain can be indicative for a first disease-related attack or a relapse of MOGAD-related optic neuritis, or NMOSD-related myelitis, brainstem, or cerebral affection. Chronic pain syndromes occur during the disease course and comprise primarily neuropathic pain and painful tonic spasms but also spasticity-related pain, back pain, and treatment-associated pain like osteoporosis. Acute ON-related pain seems to be particularly severe in MOGAD, while chronic neuropathic pain is more severe in NMOSD. Symptomatic treatment is currently insufficient to reduce pain intensity and improve the patients' quality of life. However, disease preventative immunosuppressive agents like tocilizumab and mycophenolate mofetil have shown a positive effect on pain reduction and should be further investigated. Patient care and future research should concentrate on a multidisciplinary approach of pain management, focusing on the respective pain type.

## Author Contributions

All authors contributed equally to the submitted work, analyzed the literature, wrote the manuscript, critically reviewed and revised the manuscript, and approved the final manuscript.

## Conflict of Interest

SA received a travel grant from Celgene and speaker honoraria from Roche and Bayer. GC has received speaker honoraria from Merck Serono and Bayer unrelated to this review. FP serves on the scientific advisory board for Novartis; received speaker honoraria and travel funding from Bayer, Novartis, Biogen Idec, Teva, Sanofi-Aventis/Genzyme, Merck Serono, Alexion, Chugai, MedImmune, and Shire; is an academic editor for PLoS ONE; is an associate editor for Neurology^®^ Neuroimmunology and Neuroinflammation; consulted for SanofiGenzyme, Biogen Idec, MedImmune, Shire, and Alexion; and received research support from Bayer, Novartis, Biogen Idec, Teva, Sanofi-Aventis/Genzyme, Alexion, Merck Serono, German Research Council, Werth Stiftung of the City of Cologne, German Ministry of Education and Research, Arthur Arnstein Stiftung Berlin, EU FP7 Framework Program, Arthur Arnstein Founda-tion Berlin, Guthy Jackson Charitable Foundation, and National Multiple Sclerosis of the USA.
